# Study on the potential active components and molecular mechanism of Xiao Huoluo Pills in the treatment of cartilage degeneration of knee osteoarthritis based on bioinformatics analysis and molecular docking technology

**DOI:** 10.1186/s13018-021-02552-w

**Published:** 2021-07-17

**Authors:** Weijian Chen, Tianye Lin, Qi He, Peng Yang, Gangyu Zhang, Fayi Huang, Zihao Wang, Hao Peng, Baolin Li, Du Liang, Haibin Wang

**Affiliations:** 1 Guangzhou University of Chinese Medicine, Guangzhou, 510405 Guangdong China; 2 Guangzhou Orthopedic Hospital, Guangzhou University of Chinese Medicine, Guangzhou, 510045 Guangdong China; 3 The Lab of Orthopaedics of Chinese Medicine of Lingnan Medical Research Center, Guangzhou University of Chinese Medicine, Guangzhou, ,510405 Guangdong China; 4 The First Clinical Medical College, Guangzhou University of Chinese Medicine, Guangzhou, 510405 Guangdong China; 5 Department of Joint Orthopaedic, the Third Affiliated Hospital, Guangzhou University of Chinese Medicine, Guangzhou, 510405 Guangdong China; 6 Queen’s University Belfast, University Road, Belfast, Northen Ireland BT7 1NN United Kingdom; 7 Department of Orthopaedics, Guangzhou Orthopedic Hospital, Guangzhou University of Chinese Medicine, Guangzhou, Guangdong China; 8 Department of Orthopaedics, The First Affiliated Hospital, Guangzhou University of Chinese Medicine, Guangzhou, Guangdong China

**Keywords:** Knee osteoarthritis, XHLP, Bioinformatics, Network pharmacology, Molecular docking

## Abstract

**Background:**

Knee osteoarthritis is a common joint degenerative disease. Xiao Huoluo Pills (XHLP) has been used to treat degenerative diseases such as osteoarthritis and hyperosteogeny. However, XHLP’s specific effective ingredients and mechanism of action against osteoarthritis have not been explored. Therefore, bioinformatics technology and molecular docking technology are employed in this study to explore the molecular basis and mechanism of XHLP in the treatment of knee osteoarthritis.

**Methods:**

Public databases (TCMSP, Batman-TCM, HERB, DrugBank, and UniProt) are used to find the effective active components and corresponding target proteins of XHLP (screening conditions: OB > 30%, DL ≥ 0.18). Differentially expressed genes related to cartilage lesions of knee osteoarthritis are obtained based on the GEO database (screening conditions: adjust *P* value < 0.01, |log_2_ FC|≥1.0). The Venn package in R language and the BisoGenet plug-in in Cytoscape are adopted to predict the potential molecules of XHLP in the treatment of knee osteoarthritis. The XHLP-active component-target interaction network and the XHLP-knee osteoarthritis-target protein core network are constructed using Cytoscape software. Besides, GO/KEGG enrichment analysis on core genes is performed using the Bioconductor package and clusterProfiler package in the R language to explain the biological functions and signal pathways of the core proteins. Finally, molecular docking is performed through software such as Vina, LeDock, Discovery Studio 2016, PyMOL, AutoDockTools 1.5.6, so as to verify the binding ability between the active components of the drug and the core target protein.

**Results:**

XHLP has been screened out of 71 potentially effective active compounds for the treatment of OA, mainly including quercetin, Stigmasterol, beta-sitosterol, Izoteolin, and ellagic acid. Knee osteoarthritis cartilage lesion sequencing data (GSE114007) was screened out of 1672 differentially expressed genes, including 913 upregulated genes and 759 downregulated genes, displayed as heat maps and volcano maps. Besides, 33 core target proteins are calculated by Venn data package in R and BisoGenet plug-in in Cytoscape. The enrichment analysis on these target genes revealed that the core target genes are mainly involved in biological processes such as response to oxygen levels, mechanical stimulus, vitamin, drug, and regulation of smooth muscle cell proliferation. These core target genes are involved in signaling pathways related to cartilage degeneration of knee osteoarthritis such as TNF signaling pathway and PI3K-Akt signaling pathway. Finally, the molecular docking verification demonstrates that some active components of the drug have good molecular docking and binding ability with the core target protein, further confirming that XHLP has the effect of inhibiting cartilage degeneration in knee osteoarthritis.

**Conclusions:**

In this study, based on the research foundation of bioinformatics and molecular docking technology, the active components and core target molecules of XHLP for the treatment of cartilage degeneration of knee osteoarthritis are screened out, and the potential mechanism of XHLP inhibiting cartilage degeneration of knee osteoarthritis is deeply explored. The results provide theoretical basis and new treatment plan for XHLP in the treatment of knee osteoarthritis.

## Introduction

The characteristic case changes of knee osteoarthritis (KOA) include articular cartilage degeneration, subchondral bone reactivity, osteophyte formation at the joint edge, synovial lesions, ligament laxity, and contracture [1, 2]. With the aging of the population, the incidence of knee osteoarthritis is increasing year by year. Globally, osteoarthritis affects approximately 250 million people [3]. Chronic pain and disability associated with osteoarthritis (OA) can lead to anxiety, depression, and suicidal emotions [4]. However, long-term use of non-steroidal anti-inflammatory drugs in OA patients is associated with gastrointestinal and cardiovascular side effects [5, 6]. Advanced KOA is mainly treated by joint replacement surgery, which is expensive and has many postoperative complications [7, 8]. Therefore, it is necessary to further explore the occurrence and development mechanism of KOA, and provide new theoretical basis and diagnosis and treatment guidelines for the precise treatment of KOA.

Traditional Chinese medicine has been used to treat KOA for many years. Starting from the local application of knee joint medicine and the whole body, it can effectively relieve pain symptoms, improve joint function, and protect joint structure [9, 10]. XHLP comes from the classic traditional Chinese medicine “Taiping Huimin He Jifang Prescription,” composed of six herbs: Chuanwu, Caowu, Dilong, Araceae, Frankincense, and Myrrh [11]. At present, it is mainly used to treat diseases such as rheumatoid arthritis, hyperosteosis, osteoarthritis, and stroke hemiplegia [12]. Modern pharmacological studies have confirmed that XHLP has the effects of anti-inflammatory, anti-oxidant damage, analgesia, blood vessel expansion, inhibiting platelet aggregation, and improving immunity [13]. Besides, the diester-type diterpene alkaloids of the active extracts of Radix Aconiti and Radix Aconitum play anti-inflammatory effects by inhibiting the process of prostaglandin metabolism [14] and chemotaxis of leukocytes mediated by chemokines [15]. Seo et al. [16] revealed that the antibacterial peptide lumbricusin of earthworm extract can significantly reduce the inflammatory reaction in vivo and in vitro caused by LPS such as COX-2, IL-6, and TNF-α [17]. By inhibiting the activation of neutrophil product NF-κB, frankincense extract can downregulate the expression of inflammatory factors TNF-α, IL-1, and INF-γ, alleviating inflammation [18, 19]. Meanwhile, it can inhibit the activity of collagenase MMP-1 and alleviate cartilage degeneration [20]. Therefore, XHLP is expected to have a strong anti-osteoarthritis effect. However, the potential pharmacological mechanism of XHLP and its interaction with osteoarthritis-related targets and pathways still need to be further investigated.

**Fig. 1:**
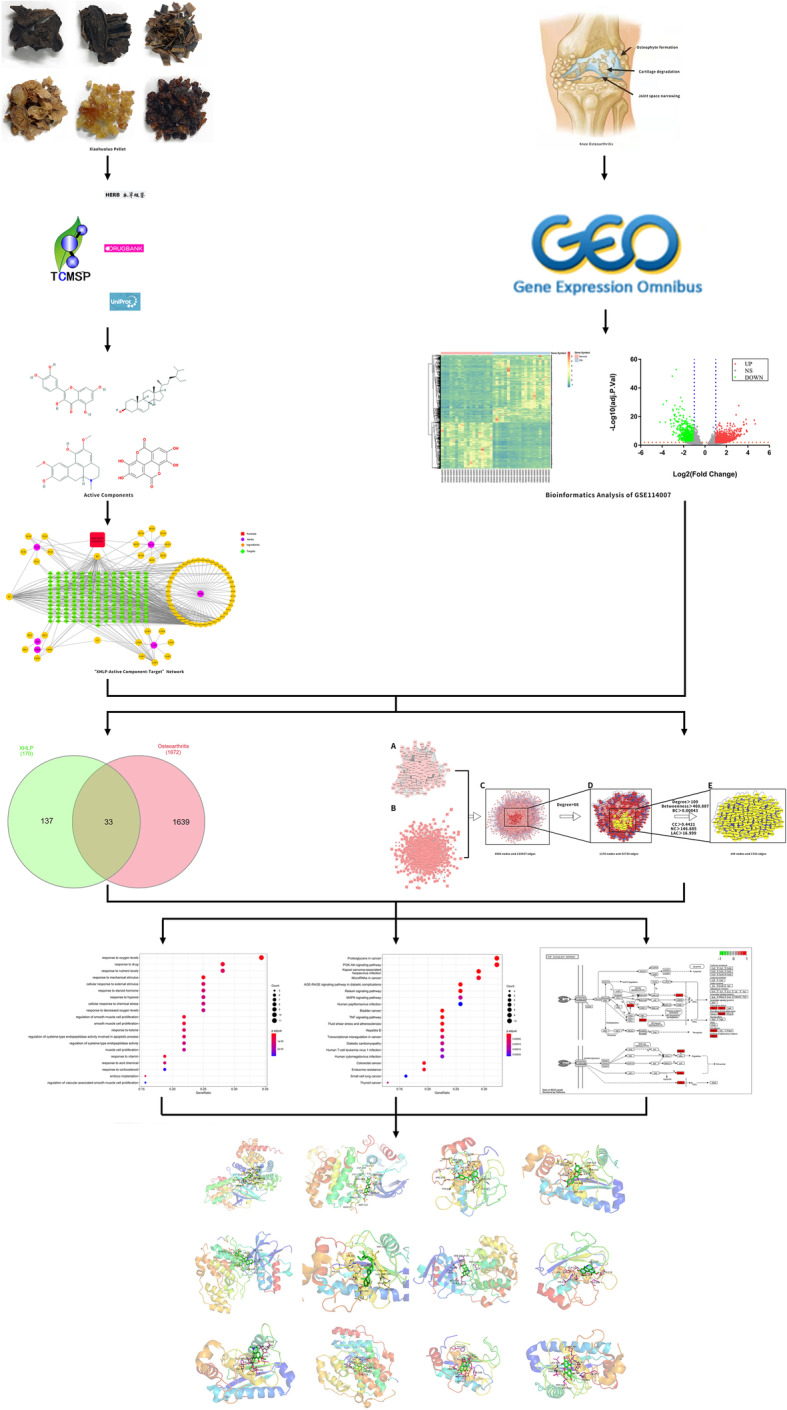
Schematic diagram for identifying the mechanism of XHLP anti-osteoarthritis by bioinformatics analysis

The traditional Chinese herbal medicine treatment KOA has passed the test of long-term medical practice. However, the “multi-component” and “multi-functional” of Chinese medicine has not been recognized by the world because its specific mechanism of action remains unclear [21]. Network pharmacology can reveal the complex network relationship between drugs, targets, and diseases through high-throughput screening, network visualization, and network topology analysis, and accurately predict and analyze the mechanism of action of traditional Chinese medicine compounds [22]. It has the characteristics of “multi-component, multi-target, multi-pathway,” turning the complex into simple. Additionally, potential differentially expressed genes are analyzed and discovered by using the genome sequencing data of KOA patients [23, 24], providing strong evidence for the biological targets and potential mechanisms of XHLP in the treatment of KOA. We speculate that the XHLP treatment of KOA through the characteristics of “multi-component, multi-target, multi-pathway.” This study aims to use bioinformatics analysis and molecular docking technology to clarify and verify the effective active components, targets, and potential mechanisms of XHLP in knee osteoarthritis (Fig. 1).

## Methods

### XHLP’s effective active components screening and identification

XHLP is composed of six traditional Chinese medicines: Chuanwu, Caowu, Dilong, Araceae, Frankincense, and Myrrh, with the composition ratio of 1.2:1.2:1.2:1.2:1.0:1.0. XHLP’s constituent medicines “Chuanwu,” “Caowu,” “Dilong,” “Araceae,” “Frankincense,” and “Myrrh” were entered into the TCM pharmacology system database [25] (TCMSP: traditional Chinese medicine system pharmacology, https://tcmspw.com/tcmsp.php) in turn for search. Oral bioavailability (OB) refers to the speed and degree of absorption of oral drugs into the circulation of the body (including the bioavailability and utilization rate of the drug). Drug likeness (DL) is a concept based on the physical and chemical properties and molecular structure of existing drugs, used to evaluate the similarity between a compound and a known drug. According to the common screening criteria of the TCMSP database and previous literature results [26, 27], the active components with OB > 30% and DL ≥ 0.18 were selected for subsequent analysis and research.

### Target protein screening and drug-active component-target network construction

The TCMSP database is used to search for the target of the active components of the drugs obtained by screening, and all the target protein names obtained from the screening of the TCMSP database are imported into the DrugBank database (https://www.drugbank.ca/) and the UniProt database (https://www.uniprot.org). The species is set as Homo sapiens. Comparison and gene name correction are performed on the obtained target protein, converting into the target gene name and deleting the target protein without corresponding gene name. The obtained active component of the medicine and the active component establish a corresponding relationship with the target. The drug-active component-target interaction network model is obtained by Cytoscape 3.7.2 software [28]. The “Networkanalyze” function in Cytoscape 3.7.2 software [29] is employed to perform network topology analysis, screening out core active components and potential target genes.

### Obtaining gene expression profile of human osteoarthritis cartilage tissue

The high-throughput sequencing data of cartilage tissue genes related to osteoarthritis are downloaded in the GEO (gene expression omnibus) database (https://www.ncbi.nlm.nih.gov/geo/). The screening conditions are (1) osteoarthritis, (2) human, and (3) cartilage tissue. The whole-genome sequencing data GSE114007 that meets the screening criteria are obtained, including 18 normal knee cartilage tissue samples from K-L I patients and 20 knee cartilage tissue samples from K-L IV KOA patients. All the above sequencing data sets are downloaded based on the GEO database of GPL11154 and GPL18573 platforms. The general clinical information of patients is presented in Table 1.

**Table 1:** Characteristics of study subjects

GSE114007	Number of subjects	Age (years, mean ± SD)	Sex		K-L stage
			Male	Female	
Normal	18	36.61 ± 13.08	13	5	I(*n* = 18)
Osteoarthritis	20	66.20 ± 7.16	8	12	IV(*n* = 20)

### Acquisition and analysis of differential genes in cartilage tissue of osteoarthritis

The original sequencing data of GSE114007 are downloaded from the GEO database. The FastQC software is adopted to perform quality assessment and quality control processing on the original data. Besides, the Hisat2 software is used to select a reference genome to compare and analyze the clean data obtained after quality control processing. Then, the number of gene reads is quantitatively analyzed using the HTSeq Python package to obtain the count file. Next, the counts data obtained by sorting are filtered. Filter conditions are (1) variance filtering and (2) low abundance filtering. Besides, the data are standardized using the Log_2_-counts per million method. The DESeq2 package in R language is employed to perform difference analysis and calculate the difference genes between the two groups. Set *P* < 0.01. adjust *P* value < 0.01 indicates that the change range is greater than or equal to twice |log_2_ FC|≥1.0 as the criterion for screening differential genes. Among them, log_2_FC≥1.0 and log_2_FC≤-1.0 represent upregulation and downregulation of gene expression, respectively. Finally, the differentially expressed genes in cartilage tissue of the osteoarthritis group and the healthy control group were derived, namely KOA differentially expressed genes (DEGs). The online analysis website ClustVi s[30] (https://biit.cs.ut.ee/clustvis/) is adopted to draw the heat map and cluster analysis of the differential genes obtained by screening. Then, the adjust *P* value in the processed data is converted to -log_10_. According to log_2_ FC, -log_10_ (adjust *P* value) is divided into upregulated genome, downregulated genome, no statistical difference, and non-expressed difference genome. The processed data are imported into GraphPad Prism 7 to draw a volcano map.

### Intersection and interactive network construction of active component of medicine and osteoarthritis target

The Venn diagram of the common target genes in the target of the active component of the drug and the target of the disease is displayed through the online Venn diagram production website InteractiVenn (http://www.interactivenn.net/) [31]. The obtained common target genes are imported into the online analysis website STRING [32] to construct protein-protein interaction (PPI). Besides, the BisoGenet plug-in in the Cytoscape 3.7.2 software [33] is used to construct the target PPI of the drug active component and the PPI network of the osteoarthritis differential gene. The “Networkanalyze” function in Cytoscape 3.7.2 software is used for network topology analysis. The PPI and KOA differential gene PPI networks of the active pharmaceutical ingredient are combined to obtain the PPI network shared by the active pharmaceutical ingredient PPI and the KOA differential gene PPI network. The CytoNCA plug-in in the Cytoscape 3.7.2 software is adopted to calculate the multi-center network topology for the PPI shared by the target of the active component of the drug and the KOA differential gene. Then, screening is conducted according to the median value of Degree, BetweennessCentrality (BC), ClosenessCentrality (CC), LAC, and NeighborhoodConnectivity (NC) values.

### Enrichment analysis of target gene by GO/KEGG

To further explore the potential mechanism of XHLP active components on KOA and the function of common target genes, we use the Bioconductor package and clusterProfiler package [34] in the R language to perform gene ontology (GO) and KEGG (Kyoto Encyclopedia of Genes and Genomes) signal pathway analysis on the target genes obtained in the previous step. The GO analysis mainly includes cellular component (CC), molecular function (MF), and biological process (BP) of potential target genes. Meanwhile, the Pathview package is used to draw the corresponding signal path diagram.

### Molecular docking verification of the binding of the active component of the drug to the target protein

The molecular docking verification of XHLP and potential targets is conducted to explore the interaction between them. The structural formula of the active components of XHLP is downloaded from the TCMSP database (https://tcmspw.com/tcmsp.php). The Chem3D software is used to make 3D structures of effective active components. Then, the 3D structure of the core protein gene is downloaded from the PDB database (http://www.rcsb.org/). The PyMOL software is employed to perform operations such as dewatering and phosphate removal of proteins. Besides, the AutoDockTools 1.5.6 software [35] is adopted to convert the pdb format of active components and core protein gene files into pdbqt format and search for active pockets. Finally, the Vina script [36] and LeDock software [37] are applied to calculate the molecular binding energy and display the results of molecular docking. Simultaneously, Discovery Studio 2016 is used to find the docking site and calculate the LibDockScore [38, 39]. Next, the output molecular docking results are imported into PyMOL software for molecular docking verification display. Binding energy < 0 indicates that the ligand and the receptor can bind spontaneously. The binding energy ≤− 5.0 kcal mol^−1^ is selected as the screening basis to evaluate the reliability of biological information analysis and prediction [40].

## Results

### Screening of effective active components of XHLP

A total of 568 active components of XHLP were retrieved through TCMSP database and Batman-TCM database, including 12 species of Radix Aconiti, 27 species of Radix Aquilinum, 123 species of Araceae, 127 species of Frankincense, and 276 species of Myrrh. However, Earth Dragon failed to retrieve the active components in the TCMSP database and Batman-TCM database. Therefore, three active components of Earthworm were obtained through literature search and HERB (A high-throughput experiment- and reference-guided database of traditional Chinese medicine, http://herb.ac.cn/) identification by the herbal group. Under the screening conditions of OB ≥ 30% and DL ≥ 0.18, 71 effective active compounds were selected from the active components of XHLP, including 3 species of Radix Aconiti and 8 species of Radix Aconiti, 3 species of Earth Dragon, 7 species of Araceae, 8 species of Frankincense, and 45 species of Myrrh; besides, there is 1 common active component of Radix Aconiti and Radix Aquilinum, and 2 common active components of Araceae and Myrrh (Table 2).

**Table 2:** XHLP effective active component information

ID	Pubchem CId	Component	OB (%)	DL	Herbs (Latin name)
CHW1	22212681	1-[(5R,8R,9S,10S,12R,13S,14S,17S)-12-hydroxy-10,13-dimethyl-2,3,4,5,6,7,8,9,11,12,14,15,16,17-tetradecahydro-1H-cyclopenta[a]phenanthren-17-yl]ethanone	33.47	0.42	Aconiti Radix
CHW2	92979	Delta4,16-Androstadien-3-one	37.63	0.31	
CAW1	133323	Izoteolin	39.53	0.51	Aconitum Kusnezoffii Reichb
CAW2	441742	Karakoline	51.73	0.73	
CAW3	21598997	3-Deoxyaconitine	30.96	0.24	
CAW4	21599000	3-Acetylaconitine	37.05	0.2	
CAW5	157539	Crassicauline A	34.13	0.21	
CAW6	155569	yunaconitine	33.56	0.2	
CAW7	441749	Napelline	34.48	0.72	
DIL1	–	Lumbrifebrine	–	–	Pheretima
DIL2	–	Lumbritin	–	–	
DIL3	–	Lumbrolysin	–	–	
TLX1	22524484	[(2R)-2-[[[(2R)-2-(benzoylamino)-3-phenylpropanoyl]amino]methyl]-3-phenylpropyl] acetate	38.88	0.56	Arisaematis Rhizoma
TLX2	5283637	24-epicampesterol	37.58	0.71	
TLX3	12303645	sitosterol	36.91	0.75	
TLX4	5997	CLR	37.87	0.68	
TLX5	5364473	8,11,14-Docosatrienoic acid, methyl ester	43.23	0.3	
RUX1	101257	tirucallol	42.12	0.75	Olibanun
RUX2	15181201	O-acetyl-α-boswellic acid	42.73	0.7	
RUX3	637234	3alpha-Hydroxy-olean-12-en-24-oic-acid	39.32	0.75	
RUX4	168928	Boswellic acid	39.55	0.75	
RUX5	44559813	Phyllocladene	33.4	0.27	
RUX6	–	3-Oxo-tirucallic,acid	42.86	0.81	
RUX7	15181201	Acetyl-alpha-boswellic,acid	42.73	0.7	
RUX8	44583885	Incensole	45.59	0.22	
MOY1	13258914	Quercetin-3-O-β-D-glucuronide	30.66	0.74	Myrrha
MOY2	5281855	Ellagic acid	43.06	0.43	
MOY3	440832	Pelargonidin	37.99	0.21	
MOY4	5283663	Poriferasta-7,22E-dien-3beta-ol	42.98	0.76	
MOY5	69232409	Guggulsterol-VI	54.72	0.43	
MOY6	128211	Mansumbinoic acid	48.1	0.32	
MOY7	57401582	Myrrhanol C	39.96	0.58	
MOY8	–	(8R)-3-oxo-8-hydroxy-polypoda -13E,17E,21-triene	44.83	0.59	
MOY9	102242792	Myrrhanones B	34.39	0.67	
MOY10	–	Epimansumbinol	61.81	0.4	
MOY11	51407984	Diayangambin	63.84	0.81	
MOY12	667495	(2R)-5,7-Dihydroxy-2-(4-hydroxyphenyl)chroman-4-one	42.36	0.21	
MOY13	–	(13E,17E,21E)-8-hydroxypolypodo-13,17,21-trien-3-one	44.34	0.58	
MOY14	–	(13E,17E,21E)-polypodo-13,17,21-triene-3,18-diol	39.96	0.58	
MOY15	–	16-Hydroperoxymansumbin-13(17)-en-3β-ol	41.05	0.49	
MOY16	–	Mansumbin-13(17)-en- 3,16-dione	41.78	0.45	
MOY17	–	(16S, 20R)-Dihydroxydammar-24-en-3-one	37.34	0.78	
MOY18	5318259	15α-Hydroxymansumbinone	37.51	0.44	
MOY19	636531	28-Acetoxy-15α-hydroxymansumbinone	41.85	0.67	
MOY20	101281214	Isofouquierone	40.95	0.78	
MOY21	11004967	[(5aS,8aR,9R)-8-oxo-9-(3,4,5-trimethoxyphenyl)-5,5a,6,9-tetrahydroisobenzofurano[6,5-f][1,3]benzodioxol-8a-yl] acetate	44.08	0.9	
MOY22	–	Phellamurin_qt	56.6	0.39	
MOY23	–	(3R,20S)-3,20-Dihydroxydammar- 24-ene	37.49	0.75	
MOY24	–	3-Methoxyfuranoguaia-9- en-8-one	35.15	0.18	
MOY25	6439929	Guggulsterone	42.45	0.44	
MOY26	441774	Petunidin	30.05	0.31	
MOY27	5280343	Quercetin	46.43	0.28	
MOY28	–	4,17(20)-(cis)-pregnadiene-3,16-dione	51.42	0.48	
MOY29	101297585	Guggulsterol IV	33.59	0.74	
MOY30	10496532	(7S,8R,9S,10R,13S,14S,17Z)-17-ethylidene-7-hydroxy-10,13-dimethyl-1,2,6,7,8,9,11,12,14,15-decahydrocyclopenta[a]phenanthrene-3,16-dione	35.75	0.48	
MOY31	–	7β,15β- dihydroxypregn-4-ene-3,16-dione	43.11	0.51	
MOY32	–	11α-hydroxypregna-4,17(20)-trans-diene-3,16-dione	36.62	0.47	
MOY33	102242791	myrrhanone A	40.25	0.63	
MOY34	–	3β-acetoxy-16β,20(R)-dihydroxydammar-24-ene	38.72	0.81	
MOY35	–	1α-acetoxy-9,19-cyclolanost-24-en-3β-ol	44.4	0.78	
MOY36	12019028	[(3R,5R,8R,9R,10R,13R,14R,17S)-17-[(2S,5S)-5-(2-hydroxypropan-2-yl)-2-methyloxolan-2-yl]-4,4,8,10,14-pentamethyl-2,3,5,6,7,9,11,12,13,15,16,17-dodecahydro-1H-cyclopenta[a]phenanthren-3-yl] acetate	33.07	0.8	
MOY37	21625900	cabraleone	36.21	0.82	
MOY38	–	(20S)-3β-acetoxy-12β,16β,25-tetrahydroxydammar-23-ene	34.89	0.82	
MOY39	–	(20S)-3β,12β,16β,25-pentahydroxydammar-23-ene	37.94	0.75	
MOY40	–	(20R)-3β-acetoxy-16β-dihydroxydammar-24-ene	40.36	0.82	
MOY41	–	3β- hydroxydammar-24-ene	40.27	0.82	
MOY42	10870920	[(5S,6R,8R,9Z)-8-methoxy-3,6,10-trimethyl-4-oxo-6,7,8,11-tetrahydro-5H-cyclodeca[b]furan-5-yl] acetate	34.76	0.25	
MOY43	–	2-Methoxyfuranoguaia-9-ene-8-one	66.18	0.18	
A1	441737	hypaconitine	31.39	0.26	Aconiti Radix/Aconitum Kusnezoffii Reichb
B1	222284	beta-sitosterol	36.91	0.75	Arisaematis Rhizoma/Myrrha
B2	5280794	Stigmasterol	43.83	0.76	Arisaematis Rhizoma/Myrrha

### Construction and analysis of XHLP-active component-target network

**Fig. 2:**
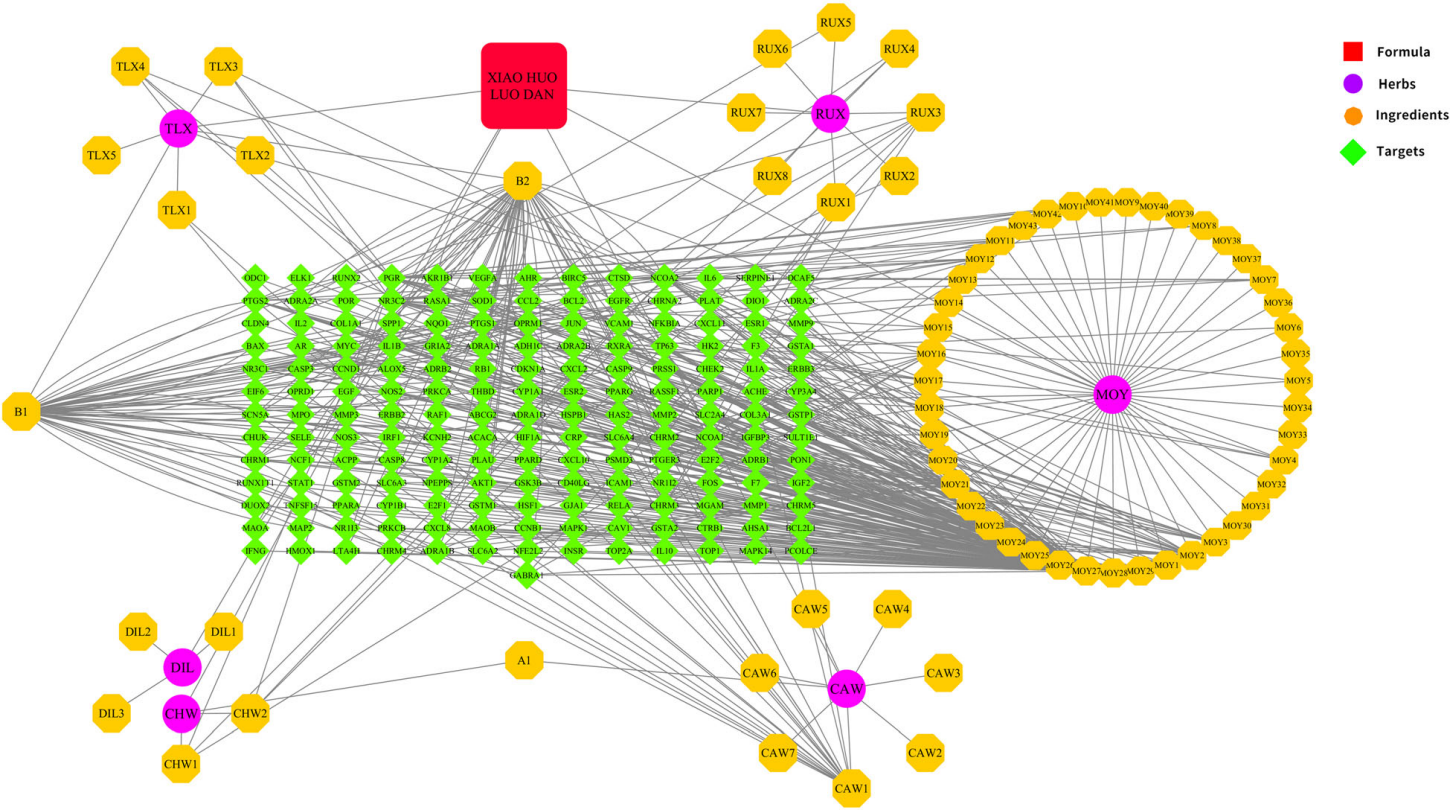
“XHLP-Active Component-Target” Network: the purple circles in the network represent the drugs in XHLP, the orange hexagons represent the active components, and the green diamonds represent the target genes

The 71 active components in XHLP were entered into the TCMSP database and Batman-TCM database to find their corresponding target proteins. The 206 target proteins obtained were imported into the DrugBank database and the UniProt database for comparison and correction, and 170 standard gene names were output. Next, the XHLP composition and effective active components and the corresponding relationship between the active components and the target was obtained. The XHLP-active component-target network was constructed using Cytoscape 3.7.2 software. The network has 248 nodes, including 6 drug nodes, 71 active compound nodes, and 170 target nodes, with a total of 506 edges (Fig. 2). Each edge in the network represents the active component contained in the drug and the interaction between the active component and the target gene. The “Networkanalyze” function in the Cytoscape 3.7.2 software is employed to perform topology analysis and calculation on the network. Among them, Degree value and BetweennessCentrality value (BC) are the key parameters to measure the nodes in the network. The active components in this network have an average relationship with 6 targets, and each target has an interconnection relationship with an average of 2.5 active components. Therefore, the active components in XHLP may be related to multiple targets while one target may be related to multiple active components. It can be revealed by analyzing the Degree value and BC value of the active component node and target gene node in the network that the top 5 active molecules are MOY27-quercetin, B2-Stigmasterol, B1-beta-sitosterol, CAW1-Izoteolin, and MOY2-ellagic acid, which can be connected to 136, 53, 53, 18, and 17 target proteins, respectively; the top six targets are PGR, NCOA2, NR3C2, PTGS2, PTGS1, and RXRA, which can interact with 25, 24, 18, 18, 12, and 12 active components, respectively (Table 3).

**Table 3:** Degree and BC values of the top 8 effective active components and targets of XHLP

Type	Description	Degree	BC	Link	Rank
**Component**	Quercetin	136	0.71368345	136	1
	Stigmasterol	52	0.08655831	52	2
	Beta-sitosterol	52	0.07340369	52	3
	Izoteolin	18	0.07627614	18	4
	Ellagic acid	17	0.03680757	17	5
	[(5aS,8aR,9R)-8-oxo-9-(3,4,5-trimethoxyphenyl)-5,5a,6,9-tetrahydroisobenzofurano[6,5-f][1,3]benzodioxol-8a-yl] acetate	16	0.03229396	16	6
	Pelargonidin	12	0.01540433	12	7
	3-Methoxyfuranoguaia-9- en-8-one	9	0.00568861	9	8
**Gene**	PGR	25	0.02750561	25	1
	NCOA2	24	0.07571674	24	2
	PTGS2	18	0.04966347	18	3
	NR3C2	18	0.00563037	18	4
	RXRA	12	0.03377059	12	5
	PTGS1	12	0.03359142	12	6
	CHRM3	10	0.00861658	10	7
	CHRM1	10	0.00440313	10	8

### Acquisition and analysis of differentially expressed genes in KOA cartilage tissue

**Fig. 3:**
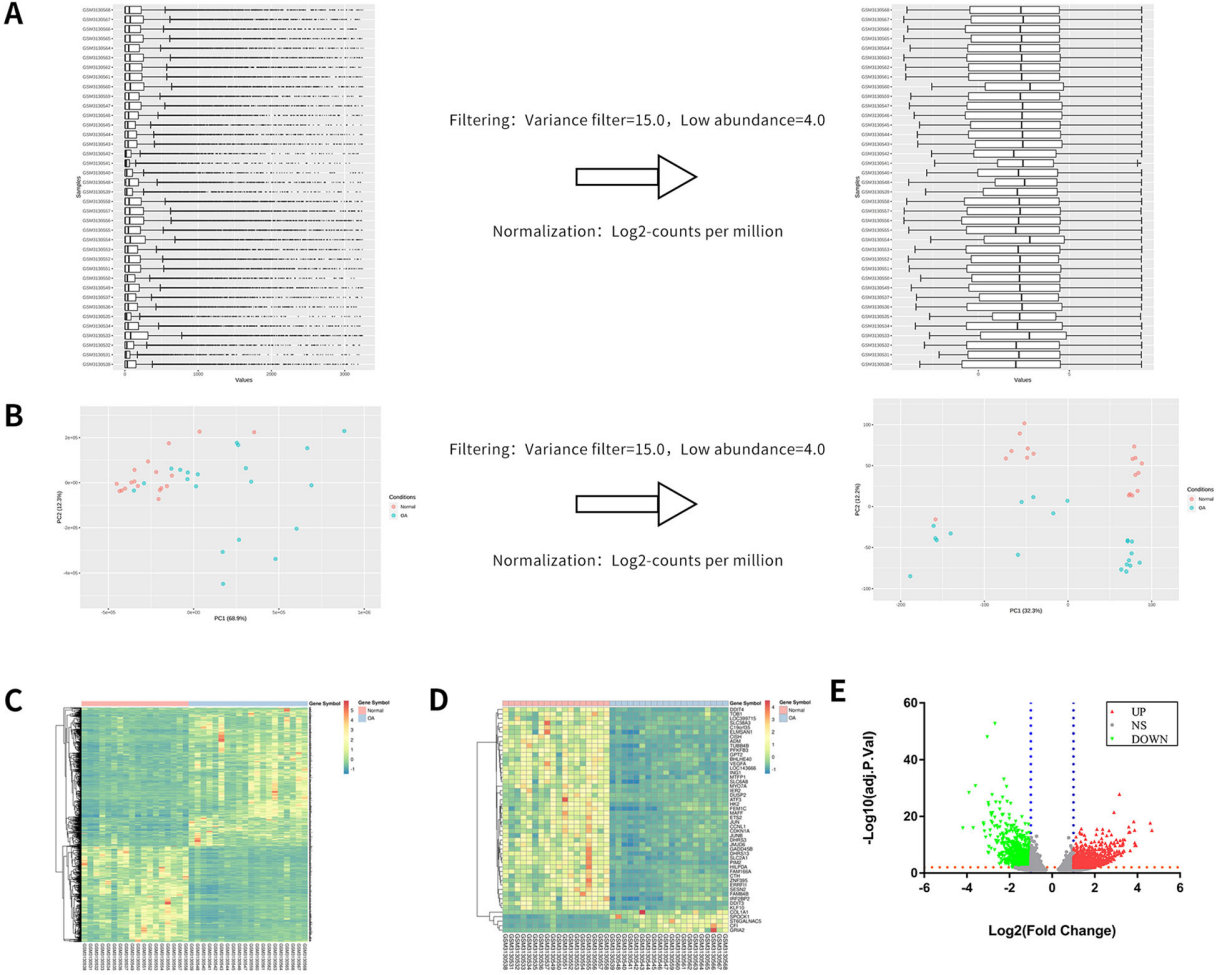
Bioinformatics analysis of GSE114007. **A** Box plots of samples before and after normalization, representing the counts distribution of each sample. **B** PCA plot before and after normalization; each point represents a sample; the farther the distance between the two samples, the greater the difference between the two samples. **C** Heat map of total 1672 DEGs in GSE114007. **D** Heat map of top 50 DEGs in GSE114007. Tissue samples are presented as columns; individual genes are presented as rows. Red indicates upregulated genes and green indicates downregulated genes in patients with KOA. The patients in the top rows in pink indicates columns were normals; the patients in the top rows in blue indicates columns were patients with KOA. **E** The volcano plot of total 1672 DEGs in GSE114007. The vertical blue lines correspond to Log_2_FC up and down and the horizontal orange line represents an adjusted *P* value of 0.01. The red point represents the differentially upregulated genes with statistical significance. The green point represents the differentially downregulated genes with statistical significance.

Our research is based on 20 cases of cartilage tissue samples from KOA patients (8 male cases, 12 female cases, aged 66.20 ± 7.16, and K-L IV patients accounted for 20 cases) and 18 cases of normal cartilage tissue samples (aged 36.61 ± 13.08, 13 male cases and 5 female cases) from the sequencing data set GSE114007. The general clinical characteristics of the patients are presented in Table 1. The filter conditions are variance filter = 15.0 and low abundance = 4.0; the standardization method is Log_2_-counts per millio. Box diagram and PCA diagram of the data filtering and before and after standardization are illustrated in Fig. 3A, B. In the GSE114007 data set, 1672 differentially expressed genes were screened, including 913 upregulated genes and 759 downregulated genes. A heat map was drawn for the DEGs selected in GSE114007 and the top 50 DEGs with the most significant differences in the adjust *P* Value selection (Fig. 3C, D). Among them, red and green represent upregulation and downregulation of gene expression. Then, -log_10_ conversion was performed on the adjust *P* value of the gene after the differential analysis process. According to log_2_ FC, -log_10_ (adjust *P* value) is divided into upregulated genome, downregulated genome, and no statistically different genome). The results were imported into GraphPad Prism 7.0 to draw a volcano map (Fig. 3E).

### XHLP effective active component-KOA intersection target analysis and mutual PPI network construction

**Fig. 4:**
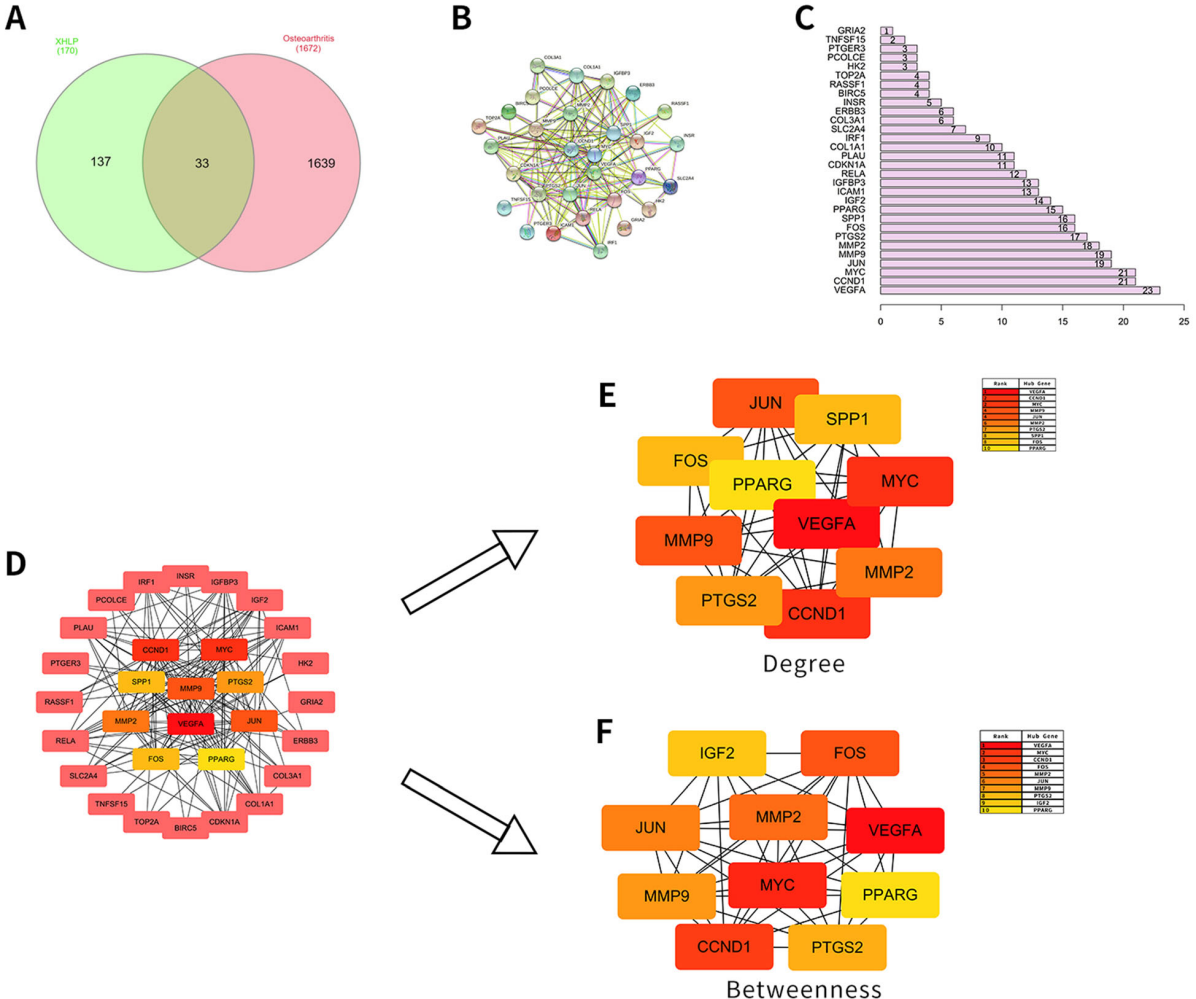
“XHLP-KOA-Common Targets” network analysis. **A** Venn diagram of KOA-related targets of XHLP into the active components. **B** PPI network generated on XHLP-KOA mapped targets. **C** Top 30 candidate targets extracted from PPI according to the node degree. **D** PPI network diagram of KOA joint targets treated with XHLP. **E** PPI network of the top 10 hub proteins of co-targets based on degree centrality. **F** PPI network of the top 10 hub proteins of co-targets based on Betweenness centrality

**Fig. 5:**
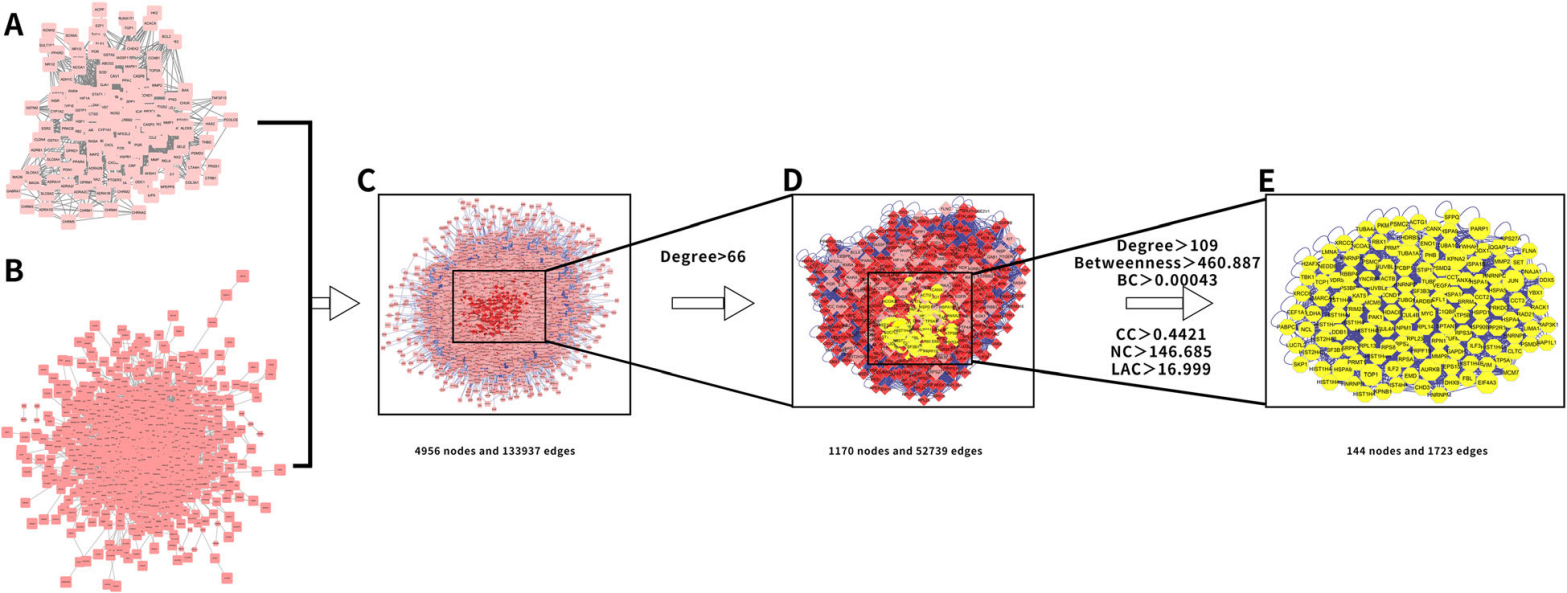
Process of topological screening for the PPI network. **A** PPI network of XHLP targets. **B** PPI network of KOA differentially expressed genes. **C** PPI network of key targets of merging mapping. **D** PPI network of key genes with a screening threshold of Degree>66 (2 times the median value of Degree). **E** PPI network of key genes with a second screening threshold of Degree>109, Betweenness>460.887, BC>0.00043, CC>0.4421, NC>146.685和LAC>16.999 (the median value of all the above)

The 170 drug active component targets obtained above and the 1672 differentially expressed genes obtained from the differential analysis of the sequencing data set GSE114007 were introduced into the online Venn diagram production website InteractiVenn. Then, a total of 33 potential targets for XHLP treatment of KOA cartilage changes were obtained by matching mapping. The Venn diagram of the effective active component-KOA intersection target is exhibited in Fig. 4A. Besides, 33 potential target genes were imported into STRING online analysis website (https://string-db.org/); hide unconnected targets were set, and “medium confidence > 0.400” in the lowest interaction score was determined; the result data of protein-protein interaction were exported (Fig. 4B, C). Next, the PPI network of potential target genes was obtained by Cytoscape 3.7.2 software, and the cytoHubba plug-in was adopted to screen Hub genes [41]. Two different algorithms, Degree and Betweenness, were employed to the top 6 potential core target genes: VEGFA, CCND1, MYC, JUN, MMP9, and MMP2 (Fig. 4D–F). Meanwhile, the BisoGenet plug-in in the Cytoscape 3.7.2 software was applied to construct the PPI network of the drug active component target and the KOA differential gene PPI network (Fig. 5A, B). The two PPI networks were merged and mapped to an intersection network (Fig. 5C). The network topology of the intersection network was analyzed and calculated using CytoNCA to obtain parameter values such as Degree, Betweenness, BetweennessCentrality (BC), ClosenessCentrality (CC), NeighborhoodConnectivity (NC), and LAC. The first screening threshold was Degree > 66 (that is, twice the median value of Degree). The result revealed that there are 1170 nodes and 52,739 edges in the secondary network graph (Fig. 5D). The second screening thresholds were Degree > 109, Betweenness > 460.887, BC > 0.00043, CC > 0.4421, NC > 146.685, and LAC > 16.999. The results indicated that there are 144 nodes and 1723 edges in the three-level network graph, which also included genes such as VEGFA, CCND1, MYC, JUN, MMP9, and MMP2 (Fig. 5E).

### Enrichment analysis of intersection target by GO/KEGG

**Fig. 6:**
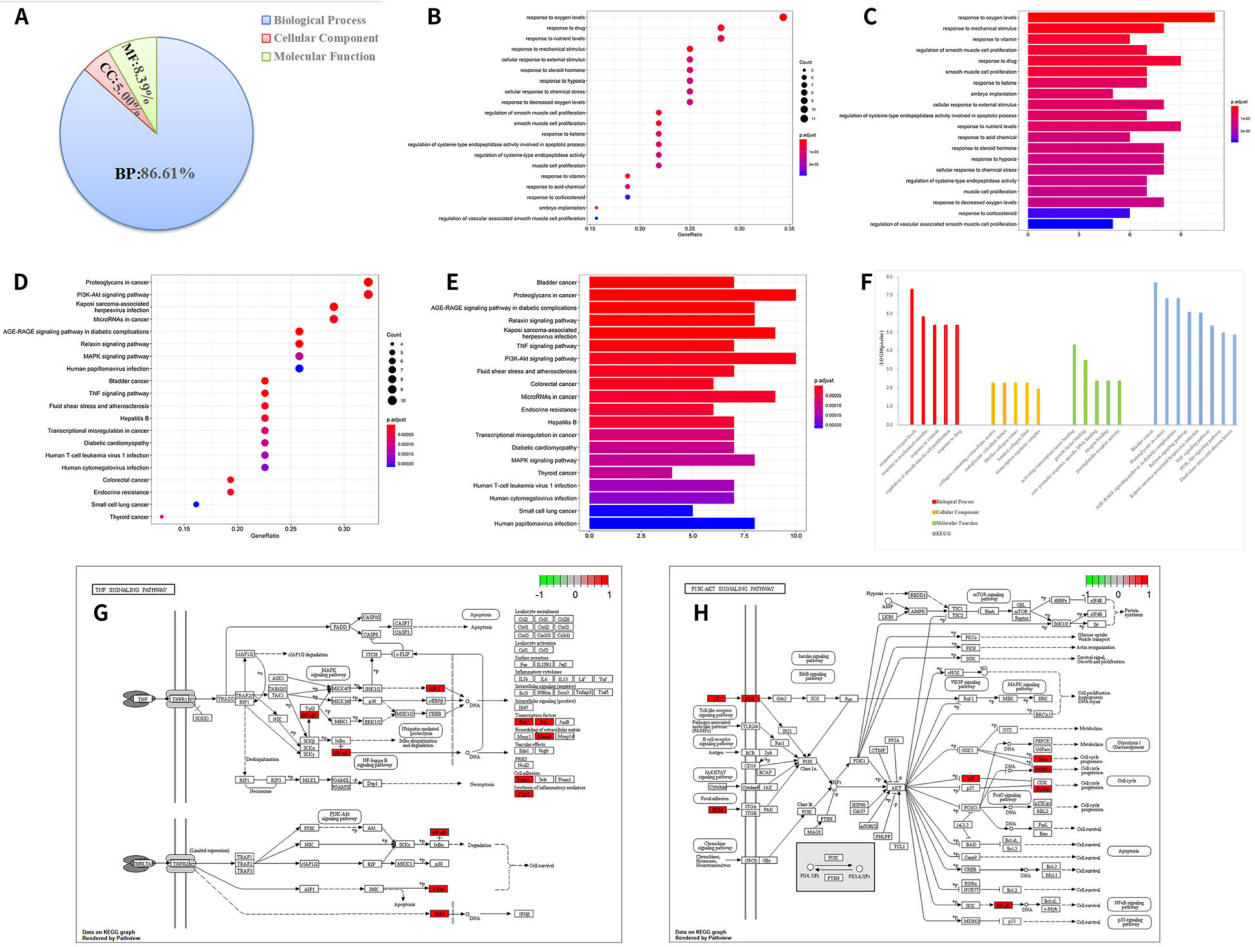
GO and KEGG enrichment analyses of 33 candidate targets. **A** Three types of GO terms for target genes and gene products. **B** The Bubble chart of the top 20 terms extracted according to the p.adjust value based on GO enrichment analysis. **C** The Bar chart of the top 20 terms extracted according to the p.adjust value based on GO enrichment analysis. **D** The Bubble chart of the top 20 pathways extracted according to the p.adjust value based on KEGG enrichment analysis. **E** The Bar graph of the top 20 pathways extracted according to the p.adjust value based on KEGG enrichment analysis. **F** Top 5 significantly enriched BP, CC, and MF categories based on gene ontology, and the Top 8 significantly enriched BP, CC, and MF categories. **G** TNF signaling pathway map of KOA targets treated with XHLP. **H** PI3K-Akt signaling pathway map of KOA targets treated with XHLP. (the red nodes represent the most potential targets, arrows represent the activation effect, T arrows represent the inhibition effect, and segments show the activation effect or inhibition effect)

The Bioconductor package and clusterProfiler package in R language were used to perform GO and KEGG pathway enrichment analysis on 33 XHLP active components-KOA intersection targets. The GO analysis of 33 potential target genes demonstrated that the biological process (BP) mainly focuses on response to oxygen levels, mechanical stimulus, vitamin, drug, and regulation of smooth muscle cell proliferation; cellular component (CC) is mainly concentrated in collagen-containing extracellular matrix, endoplasmic reticulum lumen, and fibrillar collagen trimer; molecular function (MF) is mainly manifested in activating transcription factor binding, growth factor binding, and core promoter sequence-specific DNA binding (Table 4, Fig. 6A–C). The enrichment analysis indicated that the KEGG pathway mainly focuses on AGE-RAGE signaling pathway in diabetic complications, Relaxin signaling pathway, TNF signaling pathway, PI3K-Akt signaling pathway, fluid shear stress, and atherosclerosis signaling pathways. The pathview package was adopted to display the signal pathway diagram related to cartilage (Fig 6D–G).

**Table 4:** Annotation of intersection target gene GO/KEGG enrichment analysis

Category	Term	Description	Count	P.adj. value	Genes
BP	GO:0070482	Response to oxygen levels	11	0.00000005	PTGS2,PLAU,VEGFA,CDKN1A,MMP2,PPARG,MYC,ICAM1,COL1A1,SLC2A4,HK2
	GO:0009612	Response to mechanical stimulus	8	0.00000150	PTGS2,JUN,RELA,PPARG,FOS,COL1A1,COL3A1,IRF1
	GO:0033273	Response to vitamin	6	0.00000436	PTGS2,RELA,PPARG,CCND1,COL1A1,SPP1
	GO:0048660	Regulation of smooth muscle cell proliferation	7	0.00000436	PTGS2,JUN,CDKN1A,MMP2,MMP9,PPARG,IGFBP3
	GO:0042493	Response to drug	9	0.00000436	PTGS2,RELA,CDKN1A,PPARG,CCND1,FOS,MYC,ICAM1,COL1A1
CC	GO:0062023	Collagen-containing extracellular matrix	6	0.00580234	MMP2,MMP9,ICAM1,COL1A1,COL3A1,PCOLCE
	GO:0005788	Endoplasmic reticulum lumen	5	0.00580234	PTGS2,COL1A1,COL3A1,SPP1,IGFBP3
	GO:0005583	Fibrillar collagen trimer	2	0.00580234	COL1A1,COL3A1
	GO:0098643	Banded collagen fibril	2	0.00580234	COL1A1,COL3A1
	GO:0005667	Transcription regulator complex	5	0.01219532	JUN,RELA,PPARG,CCND1,FOS
MF	GO:0033613	Activating transcription factor binding	5	0.00005068	JUN,RELA,PPARG,FOS,MYC
	GO:0019838	Growth factor binding	5	0.00035415	COL1A1,COL3A1,INSR,IGFBP3,ERBB3
	GO:0001046	Core promoter sequence-specific DNA binding	3	0.00456064	RELA,FOS,MYC
	GO:0005178	Integrin binding	4	0.00456064	IGF2,ICAM1,COL3A1,SPP1
	GO:0004955	Prostaglandin receptor activity	2	0.00456064	PPARG,PTGER3
KEGG	hsa05219	Bladder cancer	7	0.00000002	VEGFA,CDKN1A,MMP2,MMP9,CCND1,MYC,RASSF1
	hsa05205	Proteoglycans in cancer	10	0.00000015	PLAU,VEGFA,CDKN1A,MMP2,MMP9,IGF2,CCND1,MYC,COL1A1,ERBB3
	hsa04933	AGE-RAGE signaling pathway in diabetic complications	8	0.00000015	JUN,RELA,VEGFA,MMP2,CCND1,ICAM1,COL1A1,COL3A1
	hsa04926	Relaxin signaling pathway	8	0.00000087	JUN,RELA,VEGFA,MMP2,MMP9,FOS,COL1A1,COL3A1
	hsa05167	Kaposi sarcoma-associated herpesvirus infection	9	0.00000095	PTGS2,JUN,RELA,VEGFA,CDKN1A,CCND1,FOS,MYC,ICAM1
	hsa04668	TNF signaling pathway	7	0.00000479	PTGS2,JUN,RELA,MMP9,FOS,ICAM1,IRF1
	hsa04151	PI3K-Akt signaling pathway	10	0.00001120	RELA,VEGFA,CDKN1A,IGF2,CCND1,MYC,COL1A1,INSR,SPP1,ERBB3
	hsa05418	Fluid shear stress and atherosclerosis	7	0.00001428	JUN,RELA,VEGFA,MMP2,MMP9,FOS,ICAM1

### Molecular docking verification

**Fig. 7:**
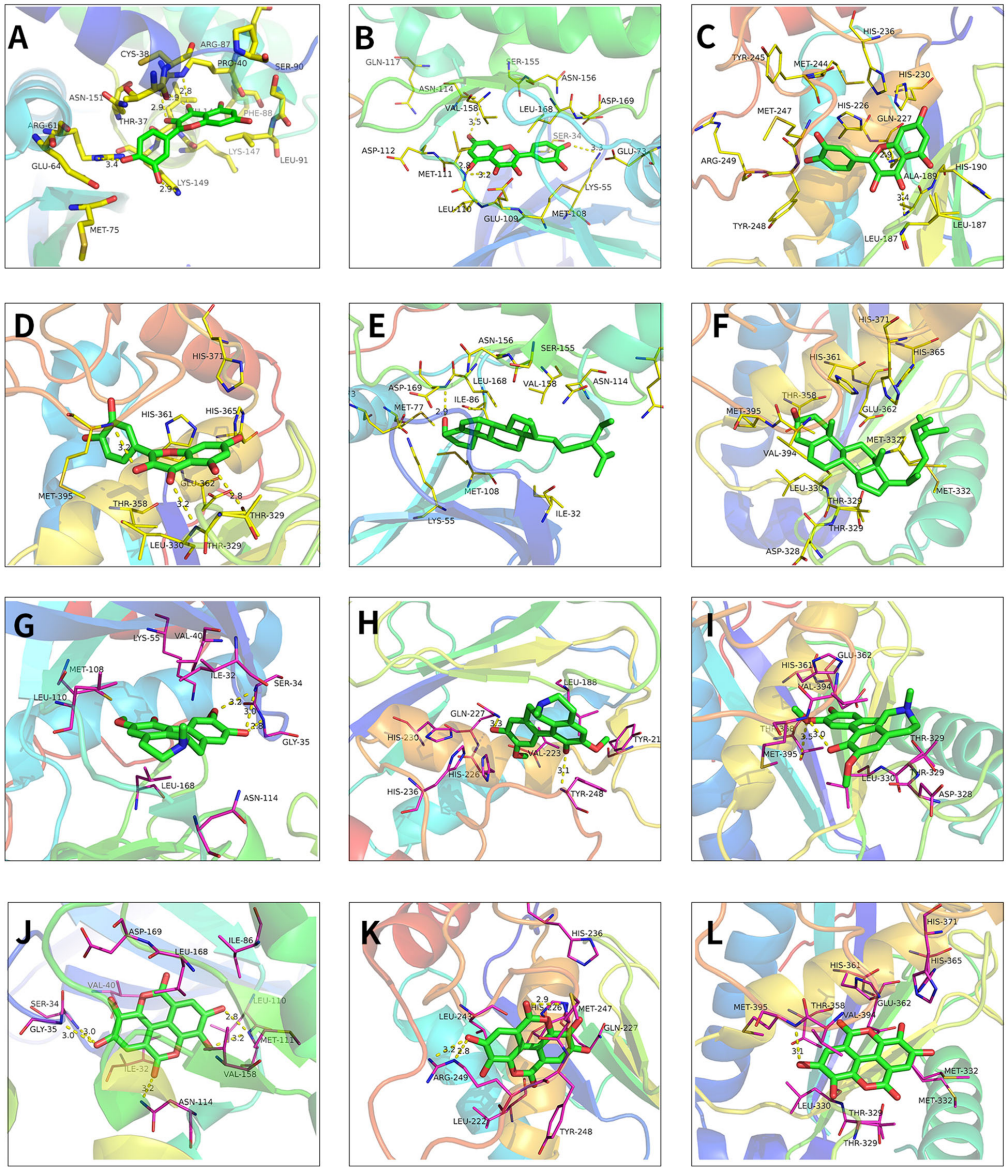
3D Molecular docking model of the 5 key active ingredients with the 6 hub-targets. **A** The molecular docking of quercetin with CCND1(2W96). **B** The molecular docking of quercetin with JUN(3V3V). **C** The molecular docking of quercetin with MMP9 (6ESM). **D** The molecular docking of quercetin with MMP2(4WK7). **E** The molecular docking of beta-sitosterol with JUN(3V3V). **F** The molecular docking of beta-sitosterol with MMP2(4WK7). **G** The molecular docking of Izoteolin with JUN(3V3V). **H** The molecular docking of Izoteolin with MMP9(6ESM). **I** The molecular docking of Izoteolin with MMP2(4WK7). **J** The molecular docking of ellagic acid with JUN(3V3V). **K** The molecular docking of ellagic acid with MMP9(6ESM). **L** The molecular docking of ellagic acid with MMP2(4WK7)

**Fig. 8:**
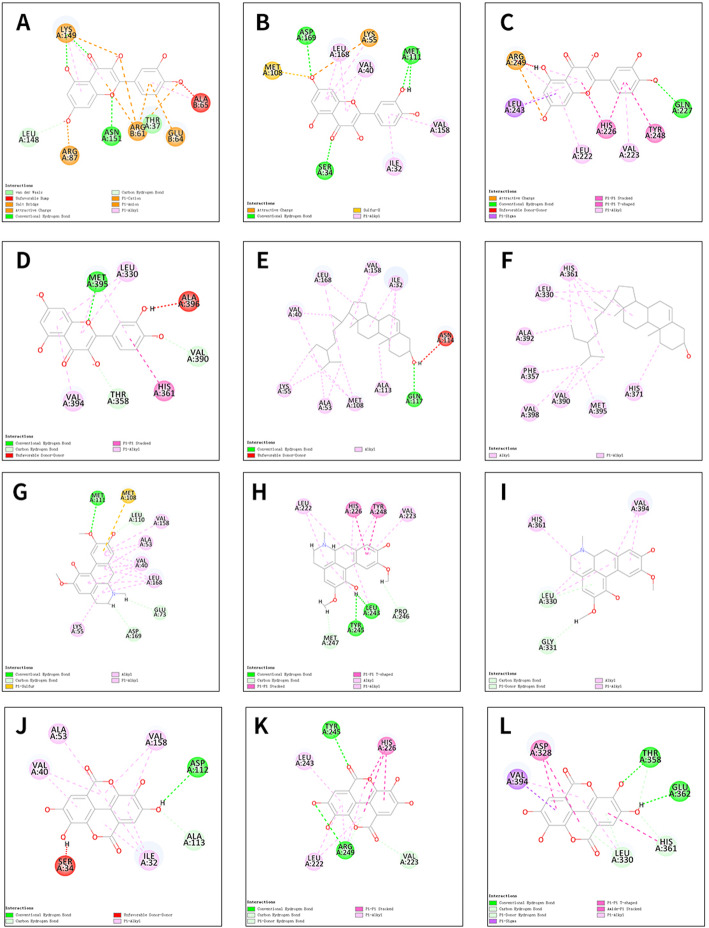
2D virtual molecular docking of bioactive ingredients from XHLP for knee osteoarthritis targets. **A** The molecular docking of quercetin with CCND1(2W96). **B** The molecular docking of quercetin with JUN(3V3V). **C** The molecular docking of quercetin with MMP9(6ESM). **D** The molecular docking of quercetin with MMP2(4WK7). **E** The molecular docking of beta-sitosterol with JUN(3V3V). **F** The molecular docking of beta-sitosterol with MMP2(4WK7). **G** The molecular docking of Izoteolin with JUN(3V3V). **H** The molecular docking of Izoteolin with MMP9(6ESM). **I** The molecular docking of Izoteolin with MMP2(4WK7). **J** The molecular docking of ellagic acid with JUN(3V3V). **K** The molecular docking of ellagic acid with MMP9(6ESM). **L** The molecular docking of ellagic acid with MMP2(4WK7)

The top 5 active molecules in the 2.2 results (quercetin, Stigmasterol, beta-sitosterol, Izoteolin, and ellagic acid) and the top 6 potential core target genes in the 2.4 results (VEGFA, CCND1, MYC, JUN, MMP9, and MMP2) were selected for molecular docking verification. The Chem3D software was employed to draw the corresponding 3D structure according to the structural formulas of the 5 effective active components, and output in mol*2 format. The 3D structures of 6 core proteins were downloaded from the PDB database and output in pdb format. The AutoDockTools 1.5.6 software was used to convert active components and core proteins into pdbqt format to find active pockets, that is, the ligand is combined with one or more amino acid residues to form H bonds, H-π bonds, H-π bonds, or π-π bonds and other active sites. Vina script, LeDock, and Discovery Studio 2016 software were operated to calculate the binding energy of ligand and receptor. The results suggested that quercetin can form a stable docking model with CCND1, JUN, MMP9, and MMP2, respectively; beta-sitosterol can dock with JUN and MMP2 protein ligands; izoteolin and ellagic acid can form stable docking with JUN, MMP9, and MMP2 protein ligands (Table 5). When the receptor-ligand binding energy calculated by Vina and LeDock was ≤− 5.0 kcal mol^−1^ and DS can find the docking site, the results of the active component receptor output by the LeDock software were imported into Pymol, and the 3D molecular docking display was performed with the protein ligand (Fig. 7). Simultaneously, the Discovery Studio 2016 software was adopted to display the results of molecular docking that meet the requirements in two dimensions (Fig. 8).

**Table 5:** Comparison of binding energy of molecular docking Vina, LeDock, and DS

Compound	Structure	Target (Ligand)	Binding energy		
			Vina (kcal mol^−1^)	LeDock (kcal mol^−1^)	DS (LibDockScore)
**Quercetin**		VEGFA(5T89)	− 6.00	− 5.09	–
		CCND1(2W96)	− 6.30	− 5.59	90.3571
		MYC(5I42)	− 8.00	− 7.70	–
		JUN(3V3V)	− 8.80	− 6.00	99.4514
		MMP9(6ESM)	− 9.90	− 6.86	145.044
		MMP2(4WK7)	− 9.50	− 6.82	138.262
**Stigmasterol**		VEGFA(5T89)	− 4.90	− 4.08	–
		CCND1(2W96)	− 6.50	− 4.00	87.8871
		MYC(5I42)	− 6.40	− 5.38	–
		JUN(3V3V)	− 7.20	− 4.86	107.282
		MMP9(6ESM)	− 8.50	− 5.12	–
		MMP2(4WK7)	− 5.80	− 4.98	117.641
**Beta**− **sitosterol**		VEGFA(5T89)	− 5.70	− 4.25	–
		CCND1(2W96)	− 6.40	− 4.27	–
		MYC(5I42)	− 6.30	− 5.53	–
		JUN(3V3V)	− 9.50	− 5.23	109.886
		MMP9(6ESM)	− 8.80	− 5.57	–
		MMP2(4WK7)	− 7.40	− 5.14	124.419
**Izoteolin**		VEGFA(5T89)	− 5.50	− 4.02	–
		CCND1(2W96)	− 5.70	− 4.70	–
		MYC(5I42)	− 6.10	− 5.95	–
		JUN(3V3V)	− 8.80	− 5.89	96.172
		MMP9(6ESM)	− 7.70	− 5.27	117.762
		MMP2(4WK7)	− 8.30	− 5.86	118.482
**Ellagic acid**		VEGFA(5T89)	− 6.10	− 4.38	–
		CCND1(2W96)	− 6.30	− 4.92	–
		MYC(5I42)	− 6.30	− 6.44	–
		JUN(3V3V)	− 9.50	− 6.40	100.529
		MMP9(6ESM)	− 7.70	− 5.47	132.727
		MMP2(4WK7)	− 8.60	− 5.80	120.031

## Discussion

Bioinformatics was first proposed by Dr. Li Huaan in 1978. People gradually realized the importance of combining computer science with biology. With the continuous surge of experimental data and biological information (about doubling every 15 months), information management, and analysis has become an urgent problem to be solved. Therefore, data mining can help us find valuable information from massive amounts of data and convert it into meaningful knowledge. At present, life science research is transforming from experimental analysis combined with data accumulation to data analysis to guide experimental verification [42]. Network pharmacology and bioinformatics analysis can be based on massive experimental data and clinical trial results, contributing to exploring the potential mechanism of TCM treatment and prevention of diseases, especially for the research on the complex chemical components of Chinese herbal medicine and the multi-target and multi-mechanism of diseases [43]. Studies have discovered that some of the extracts in the XHLP composition of drugs play an essential role in the treatment of KOA. However, the specific treatment mechanism has not been thoroughly studied. Therefore, based on bioinformatics analysis and mining, this study explored the potential effective active components, molecular targets, and mechanism of action of XHLP in the treatment of KOA cartilage degeneration, and further used molecular docking technology to verify the binding ability of the effective active components in XHLP with molecular targets. It fully verified the molecular basis of XHLP treatment of KOA and provided a theoretical basis for clinical treatment of KOA. Besides, bioinformatics analysis demonstrated that the potential mechanism of XHLP in the treatment of KOA cartilage degeneration has the features of “multi-component, multi-target, multi-pathway.”

In this study, the XHLP-active component-target network was constructed. The network topology analysis revealed that the potential active components of XHLP treatment KOA mainly include quercetin, Stigmasterol, beta-sitosterol, Izoteolin, and ellagic acid. Hu et al. [44] discovered that quercetin can inhibit the inflammation and apoptosis of chondrocytes and provide cartilage cells with an environment promoting cartilage formation to enhance cartilage repair in the OA environment. Gabay [45] observed that Stigmasterol is a plant sterol that binds to chondrocyte membranes, can reduce the expression of MMP related to cartilage degradation and the pro-inflammatory mediator PGE2 by inhibiting the NF-κB pathway, and has the properties of anti-inflammatory and inhibiting cartilage matrix catabolism. KIM et al. [46] proposed that in high-fat diet-induced intestinal inflammation, beta-sitosterol can inactivate NF-κB by interfering with the binding between LPS and TLR4 to reduce the production of pro-inflammatory cytokines. Besides, Lin et al. [47] demonstrated that ellagic acid can inhibit inflammation caused by IL-1β stimulation; reduce the expression of MMP-13, ADAMTS5, COX-2, and INOS in human OA chondrocytes; and weaken IL-1β-induced inflammation by inhibiting the NF-κB pathway. There is no related research on Izoteolin in KOA. Therefore, the effective active components in XHLP may inhibit cartilage cell apoptosis, reduce cartilage matrix catabolism, and impede the NF-κB pathway to lower the production of pro-inflammatory cytokines, playing a synergistic effect in slowing down the progression of KOA cartilage degeneration.

Through the mutual mapping of the XHLP target and the differentially expressed genes of KOA, 33 potential molecular targets were obtained. Furthermore, Cytohubba, BisoGenet, and CytoNCA plug-in in Cytoscape software were used to obtain 6 potential therapeutic targets for XHLP treatment of KOA, namely VEGFA, CCND1, MYC, JUN, MMP9, and MMP2. A large number of studies have revealed that the expression of VEGFA is elevated in patients with advanced OA [48–50]. VEGFA can upregulate the expression of MMP-1, MMP-3, and MMP-13 in chondrocytes and downregulate the expression of key extracellular matrix components (including aggrecan and type II collagen) to accelerate the process of KOA cartilage degeneration. Zan et al. [51] discovered that CCND1 gene silencing can enhance the inhibitory effect on IL-1β-induced proliferation of OA chondrocytes. Zou et al. [52] put forward that silencing the C-myc gene can reduce the effect of IL-1β on the cycle progression and apoptosis of chondrocytes in OA rats. Rhee et al. [53] found that IL-1β activates the expression of JUN in articular chondrocytes, inhibiting the expression of SOX9 and type II collagen in chondrocytes and the anabolic reaction of the extracellular matrix, accelerating the damage of arthritic chondrocytes. Studies have suggested that MMP-9 exacerbates local inflammation by increasing the expression of pro-inflammatory mediators such as TNF-a and IL-1, leading to accelerated degeneration of cartilage and overgrowth of subchondral bone [54, 55]. Besides, MMP-2 can effectively lyse and inactivate monocyte chemotactic protein 3 (MCP-3). This not only prevents the inflammatory response from starting but also removes inflammatory factors such as TNF-a and IL-17 in the body, preventing the further development of inflammation [56]. Therefore, the above targets may all play a role in the development of KOA cartilage degeneration and are potential targets for XHLP to treat KOA.

The enrichment analysis of target genes GO/KEGG indicated that “oxygen levels,” “mechanical stimulus,” “collagen-containing extracellular matrix,” “TNF signaling pathway,” “PI3K-Akt signaling pathway,” and “Fluid shear stress” may be a potential way of XHLP treatment of KOA. Many studies [57–59] have revealed that the mechanical stress environment of joints is a significant factor affecting the activity of cartilage cells in the body, and accelerates the catabolism of osteoarthritis cartilage by interacting with pro-inflammatory factors. TNF-α can inhibit the synthesis of matrix macromolecules; stimulate the synthesis and secretion of cartilage degrading enzymes, prostaglandin (PGE2); and promote the synthesis of inflammatory factors, leading to cartilage destruction, increased inflammation, and finally degeneration of KOA cartilage [60, 61]. Moreover, the activation of PI3K/AKT signaling pathway can inhibit chondrocyte apoptosis and promote the proliferation of chondrocytes to slow down the process of KOA cartilage degeneration [62, 63]. Meanwhile, the effective active components in XHLP have the characteristics of inhibiting chondrocyte apoptosis, reducing cartilage matrix catabolism, and eliminating the production of pro-inflammatory cytokines. Therefore, XHLP can play a role in inhibiting chondrocyte apoptosis, anti-inflammatory, and other mechanisms to treat or delay the degeneration of KOA cartilage.

Finally, the binding ability of the active components of XHLP with molecular target domains was verified in this study through molecular docking technology. The results demonstrated that quercetin can form a stable docking model with CCND1, JUN, MMP9, and MMP2, respectively; beta-sitosterol can dock with JUN and MMP2 protein ligands; Izoteolin and ellagic acid can form stable docking with JUN, MMP9, and MMP2 protein ligands. According to the calculations with Vina, LeDock, and DS software, quercetin can form the most stable molecular docking with the 6ESM ligand of MMP9 protein; MMP9 is a pro-inflammatory protein, and can exert a direct anti-inflammatory effect to treat KOA cartilage degeneration when combined with quercetin, which is the effective active component in XHLP. However, this study still has some limitations. First, the results of this study lack cell and animal experimental verification. Another limitation is the false negative due to the targets rooted from a different database, which may have biased impact because of different experimental conditions. We are also aware of the public databases that have limited information. Moreover, Earth Dragon failed to retrieve the active components in the TCMSP database and Batman-TCM database.

## Conclusion

In this study, the potentially effective active components, molecular targets, and action pathways of XHLP in the treatment of KOA cartilage degeneration were analyzed using bioinformatics. Besides, 71 effective active components were discovered for potential treatment of KOA; 1672 differentially expressed genes in cartilage degeneration with KOA were discovered through bio-information analysis. Mutual mapping was used to obtain 33 potential targets for the XHLP treatment of KOA. This study discovered 33 molecular targets such as VEGFA, CCND1, MYC, JUN, MMP9, and MMP2. These molecular targets are associated with the functions of “inflammatory response,” “mechanical stimulus,” “TNF signaling pathway,” and “PI3K-Akt signaling pathway,” involved in signaling pathways related to inflammation and chondrocyte apoptosis. Moreover, molecular docking verification was conducted to verify that the active components of XHLP can form a stable docking model with potential molecular targets. To sum up, the XHLP treatment of KOA plays a synergistic role in anti-inflammatory and inhibiting chondrocyte apoptosis through the characteristics of “multi-component, multi-target, multi-pathway.”

## Data Availability

All the data will be available upon motivated request to the corresponding author of the present paper.

## Abbreviations

OA: Osteoarthritis
KOA: Knee osteoarthritis
XHLP: Xiao huoluo pills
TCMSP: Traditional Chinese medicine system pharmacology
OB: Oral bioavailability
DL: Drug-likeness
GEO: Gene expression omnibus
DEGs: Differentially expressed genes
PPI: Protein-protein interaction
BC: Betweenness centrality
NC: Neighborhood connectivity
GO: Gene ontology
KEGG: Kyoto encyclopedia of genes and genomes
CC: Cellular component
MF: Molecular function
BP: Biological process
